# Notion of Opacity Considering Security Levels for Piecewise Affine Systems

**DOI:** 10.3390/s26123771

**Published:** 2026-06-12

**Authors:** Taiga Matsumae, Koichi Kobayashi, Yuh Yamashita

**Affiliations:** Graduate School of Information Science and Technology, Hokkaido University, Sapporo 060-0814, Japan

**Keywords:** cyber-physical systems, discrete-time piecewise affine systems, opacity, security levels

## Abstract

Cyber-physical systems (CPSs) integrate physical processes and information components through communication networks and are therefore vulnerable to cyber attacks. Opacity is a security property that prevents an adversary from inferring sensitive information from observations, and it has been studied mainly for discrete-event systems. In this paper, we extend this concept to discrete-time piecewise affine (DT-PWA) systems, which constitute an important class of hybrid systems used to model CPSs. In conventional opacity analysis, the result is typically binary, i.e., a system is either opaque or not. For systems with continuous dynamics, however, such a binary characterization may be insufficient, and it is desirable to evaluate the degree of security. To address this issue, we introduce a notion of opacity that incorporates security levels. We first formulate opacity for DT-PWA systems and then derive a necessary and sufficient condition for opacity. Based on this condition, we present a verification method using polytope computations and discuss the interpretation of the proposed notion. Finally, a numerical example is provided to illustrate the effectiveness of the proposed method.

## 1. Introduction

With the rapid development of Internet-of-Things (IoT) technologies, cyber-physical systems (CPSs) have attracted considerable attention. A CPS tightly integrates physical processes and computational components through communication networks [1,2,3,4]. In such systems, sensing, computation, communication, and control are closely coupled so that decisions made in cyberspace directly affect the behavior of physical processes, and vice versa. This tight integration has significantly improved the efficiency, autonomy, and flexibility of modern engineered systems, while also increasing their structural complexity. In addition, the large-scale interconnection of heterogeneous devices enables CPSs to support real-time monitoring, automated decision-making, and adaptive operation in dynamic environments. CPSs are now widely deployed in many application domains, including power systems [5], water distribution networks [6,7], medical devices [8,9], and home control systems [10,11]. They also play an important role in transportation, manufacturing, and other safety-critical infrastructures, where reliable operation is essential. In these applications, even a small mismatch between cyber decisions and physical behavior may lead to degraded performance, safety risks, or service interruptions. Because these systems rely on information exchange across devices and networks, they are vulnerable to cyber attacks, communication failures, and malicious manipulation of sensed or transmitted data. As a result, security problems in CPSs can propagate from the cyber layer to the physical layer, potentially causing serious physical and societal damage [12,13].

A representative example is the cyber attack on the Ukrainian power grid in December 2015. In that incident, attackers remotely accessed circuit breakers, disconnected substations, and shut down backup power sources, leaving more than 200,000 people without electricity for several hours. Similar attacks could cause substantial damage to other critical infrastructures as well [14]. Other cyber attacks on CPSs have also been reported in the literature [15,16]. These examples highlight the importance of developing security notions that prevent attackers from extracting critical information from system observations. Other notable examples include the Stuxnet worm targeting Iranian nuclear centrifuges [16], the TRITON/TRISIS attack disabling safety instrumented systems at a petrochemical plant [17], and the 2016 Industroyer malware targeting Ukrainian power infrastructure [18].

One important security notion for this purpose is opacity. Informally, opacity characterizes whether an adversary can infer sensitive information about a system state from its observations [19,20]. From a security perspective, opacity provides a formal way to describe whether confidential system information can be hidden from an external observer even when some outputs are available. This property is particularly relevant to CPSs, because attackers may exploit observed data to estimate internal states, operating modes, or other sensitive information that should remain undisclosed. Opacity has been studied extensively for discrete-event systems, where fundamental concepts were established in [21] and surveyed in, for example, [22,23,24]. In the discrete-event setting, opacity has been investigated under several formulations, including current-state opacity, initial-state opacity, and related variations, and these notions have provided useful theoretical tools for analyzing information leakage. However, many practical CPSs involve continuous-valued states, inputs, and outputs, and therefore require opacity notions beyond the discrete-event systems framework. Extending opacity analysis to such systems is important for bridging the gap between well-established theory and the characteristics of realistic CPS models.

Opacity-related notions have also been investigated for discrete-time systems with continuous states. For example, [25] developed opacity notions for discrete-time linear systems, while [26] studied related problems for stochastic systems. For CPSs, however, it is often essential to model switching behavior whose dynamics depend on the current state or operating mode. Based on [27,28], we adopt a hybrid system as a mathematical model of CPSs. A hybrid system combines continuous dynamics, represented by difference or differential equations, with discrete dynamics, often represented by finite automata. Opacity has been studied for switched systems, in which continuous dynamics switch according to an external signal [29,30], but switched systems constitute only a special class of hybrid systems. To the best of our knowledge, opacity for more general hybrid systems remains insufficiently explored.

Motivated by this gap, this paper develops an opacity notion for discrete-time piecewise affine (DT-PWA) systems. In DT-PWA systems, the state, control input, and output are generally continuous variables, and the system dynamics change according to the current state [31]. A DT-PWA system is well known as a typical mathematical model of hybrid system. Moreover, DT-PWA systems are known to be equivalent to several important classes of hybrid systems [32,33]. For this reason, we adopt DT-PWA systems as a tractable and expressive model for CPSs.

The proposed notion is based on current-state opacity [21,22]. In dynamical systems, information evolves over time, and the current state is often more relevant than the initial state from the viewpoint of security. In addition, conventional opacity analysis is essentially binary: a system either satisfies opacity or it does not. For systems with continuous dynamics, such a binary assessment may be insufficient, and it is desirable to quantify how secure a system is. To address this issue, we introduce security levels into the opacity framework. Specifically, we partition the state space into one secret set and multiple non-secret sets, assign a security level to each non-secret set, and evaluate opacity accordingly. This formulation enables a more refined security assessment than the conventional binary notion.

The main contributions of this paper can be summarized as follows.

1. The notion of opacity considering security levels for DT-PWA systems is proposed.
2. A necessary and sufficient condition for the system to be opaque is derived.
3. The proposed method is demonstrated through a numerical example.

This paper is organized as follows. In Section 2, we introduce DT-PWA systems and define the reachable set. In Section 3, we formulate opacity with security levels. In Section 4, we present the main results, including a necessary and sufficient condition for opacity, an interpretation of the proposed notion, and a verification method. In Section 5, we demonstrate the effectiveness of the proposed method through a numerical example. Finally, Section 6 concludes the paper.

**Notation:** Let R denote the set of real numbers. For a given set $X\subseteq R^{n}$, a matrix $H\in R^{m\times n}$, and a vector $f\in R^{m}$, define the set HX+f:={y∣∃x∈Xsuchthaty=Hx+f}. When *f* is the zero vector, we may write H(X) instead of HX.

## 2. Discrete-Time Piecewise Affine Systems

As a mathematical model of a controlled CPS, we consider the following DT-PWA system:

(1) $$\left\{\begin{array}{c} x\left(t+1\right)=A_{I(t)}x\left(t\right)+a_{I(t)}\;\;\;\;if\;x\left(t\right)\in S_{I(t)},\\ y(t)=Cx(t), \end{array}\right.$$

where $x\left(t\right)\in X\subset R^{n}$ is state, I(t)∈{1,2,…,M} is mode, $y\left(t\right)\in R^{p}$ is output, t=0,1,2,… is discrete time. The subset $S_{i}$ assigned to mode *i* is a polytope on $R^{n}$, and satisfies ${⋃}_{i}S_{i}=X\subset R^{n}$ and $S_{i}⋂S_{j}=\emptyset$, i≠j. The coefficient matrix $A_{I(t)}\in \left\{A_{1},A_{2},\ldots ,A_{M}\right\}$ and the affine term $a_{I(t)}\in \left\{a_{1},a_{2},\ldots ,a_{M}\right\}$ in the state equation are determined according to the state (i.e., the mode) at the current time. The coefficient matrix *C* in the output equation is given as a constant matrix that does not depend on the mode. For simplicity, we assume that $A_{1},A_{2},\ldots ,A_{M}$ are invertible.

We define two sets of the state.

**Definition** **1.** *For the system* (1)*, let Pre(Z) denote the set of states that can reach the state set Z after one time step. The set Pre(Z) is defined as*

(2) $$Pre\left(Z\right):=\overset{M}{\underset{i=1}{⋃}}\left(\left(A_{i}^{-1}Z-A_{i}^{-1}a_{i}\right)⋂S_{i}\right).$$

**Definition** **2.** *For the system* (1)*, let $P^{\left(K\right)}\left(Z\right)$ denote the set of states that can reach the state set Z after time K. The set $P^{\left(K\right)}\left(Z\right)$ is defined as*

$$P^{\left(K\right)}\left(Z\right):=\underset{K}{\underset{︸}{Pre(\cdots (Pre(Pre}}\left(Z\right)))).$$

## 3. Notion of Opacity Considering Security Levels

In this section, we consider the definition of opacity for the system (1). First, we define the set of secret states and the set of non-secret states. After that, we introduce the notion of the opacity of the system (1). Next, we explain the security levels for the opacity.

### 3.1. Definition of Opacity

First, a set of secret states and a set of non-secret states are defined by $X_{s}$ and $X_{ns}=X\setminus X_{s}$, respectively. Assume also that $X_{ns}$ can be divided into *m* sets of $X_{ns_{1}},X_{ns_{2}},\ldots ,X_{ns_{m}}$ with no overlaps. Here, the secret state is important information in the system that should not be made known to the outside, and the non-secret state represents other information.

Next, we suppose that there exists an adversary in the system (1) whose goal is to determine whether the current system state x(t) is included in the secret state set or not. We suppose that the adversary knows in advance $A_{1},A_{2},\ldots ,A_{M}$, $a_{1},a_{2},\ldots ,a_{M}$, and *C* of the system (1). We also suppose that the adversary also can observe the outputs at time t−K, where *t* is the current time and *K* is given a positive constant.

Under this condition, we give the definition of opacity in the system (1) as follows.

**Definition** **3.** *For the system* (1)*, assume that a set of secret states $X_{s}$ and sets of non-secret states $X_{ns_{p}}$, p=1,2,…,m are given. Suppose that the positive constant K is given. Suppose also that an adversary knows the coefficient matrices $A_{1},A_{2},\ldots ,A_{M},C$ of the system* (1) *and the output y(t−K) at time t−K, where t is the current time. Then, the system* (1) *is said to be p-opaque if for any state $x\left(t\right)=x^{\prime }\in X_{s}$, there exists an $x\left(t\right)=x^{\prime \prime }\in X_{ns_{p}}$ such that $y^{\prime }\left(t-K\right)=y^{\prime \prime }\left(t-K\right)=y\left(t-K\right)$, where $y^{\prime }\left(t-K\right)$ denotes the output at time t−K for $x\left(t\right)=x^{\prime }\in X_{s}$, and $y^{\prime \prime }\left(t-K\right)$ denotes the output at time t−K for $x\left(t\right)=x^{\prime \prime }\in X_{ns_{p}}$.*

In [25], opacity was studied for discrete-time linear and nonlinear systems. However, such models are not always sufficient for representing CPSs, because they do not explicitly capture logical rules or mode-switching behavior.

Furthermore, the definition in [25] is based on initial-state opacity. Under initial-state opacity, the adversary aims to determine, from the observed output at time *K*, whether the initial state belongs to the secret set $X_{s}$ or not. In contrast, Definition 3 in this paper focuses on the current state. That is, the sensitive information to be protected is the current state of the system rather than its state at time 0. Because the state of a dynamical system evolves over time, the current state is often more relevant than the initial state from the viewpoint of security. Therefore, Definition 3 is more suitable for the present setting than the definition in [25]. We remark that the notion of opacity in Definition 3 is based on the output. Hence, we do not focus on state estimation using an observer.

### 3.2. Security Levels

When the non-secret state set is close to the secret state set or is clearly far from the secret state set, the degree of security is considered to be low, even if the system becomes opaque. Therefore, we divided the non-secret state set $X_{ns}$ and determined the security levels for each divided non-secret state set. In this paper, we suppose that we set the security level to be higher as the value of *p* in *p*-opacity is higher. In other words, between 1-opacity and 2-opacity, 2-opacity has a higher security level. This high security level is assumed to be a situation in which it is more difficult for an adversary to attack the system. Specifically, we assume a situation in which the higher the security level for a certain system, the easier it is to detect an adversary’s attack by adding more data consistency checks.

The security levels proposed in this paper merely define an order for a subset of the state space. In the current stage, we do not consider properties such as a quantitative leakage measure, a monotonic relation with distinguishability, and a connection to attack detection capability. Considering these properties is one of the challenges for future research. However, the proposed method has the advantage of being more flexible than existing methods that use a binary choice between secret sets and everything else.

## 4. Main Results

In this section, we first present a necessary and sufficient condition for the system to be *p*-opaque. Next, we clarify the meaning of the proposed notion of opacity based on the definition and the obtained theorem. Third, we present a method for verifying *p*-opacity. Finally, we discuss the case of linear systems as a special case.

### 4.1. Necessary and Sufficient Condition

In this subsection, we derive a necessary and sufficient condition for the system (1) to be *p*-opaque.

**Theorem** **1.** *The system* (1) *is p-opaque if and only if the following condition holds:*

(3) $$C\left(P^{\left(K\right)}\left(X_{s}\right)\right)\subseteq C\left(P^{\left(K\right)}\left(X_{ns_{p}}\right)\right).$$

**Proof.**  First, assume that the system (1) is *p*-opaque. That is, there exists a common output at time t−K for any state at time *t* included in the secret state set $X_{s}$ and some state at time *t* included in the non-secret state set $X_{ns_{p}}$. This implies that (3) holds.

Next, assume that (3) holds. The set $C(P^{\left(K\right)}\left(X_{s}\right))$ represents the output set at time t−K where the state at time *t* is included in the secret state set $X_{s}$. In a similar way, the set $C(P^{\left(K\right)}\left(X_{ns_{p}}\right))$ represents the output set at time t−K where the state at time *t* is included in the non-secret state set $X_{ns_{p}}$. The condition (3) implies there exists the same output included in $C(P^{\left(K\right)}\left(X_{ns_{p}}\right))$, for any output included in $C(P^{\left(K\right)}\left(X_{s}\right))$. That is, the system (1) is *p*-opaque.

This completes the proof. □

### 4.2. Meaning of Opacity Proposed in This Paper

**Figure 1:**
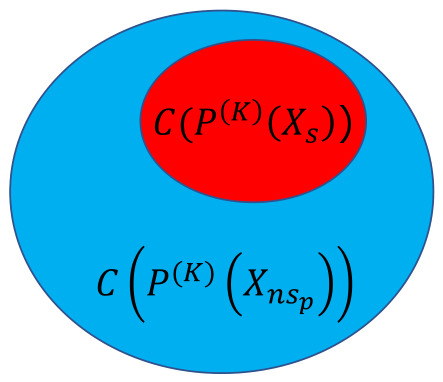
The state where $C(P^{\left(K\right)}\left(X_{s}\right))$ is completely contained in $C(P^{\left(K\right)}\left(X_{ns_{p}}\right))$.

**Figure 2:**
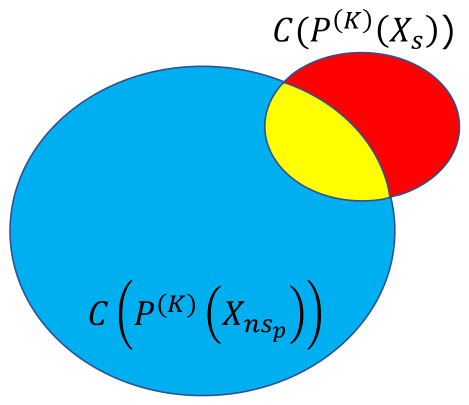
The state where $C(P^{\left(K\right)}\left(X_{s}\right))$ is not completely contained in $C(P^{\left(K\right)}\left(X_{ns_{p}}\right))$.

Figure 1 and Figure 2 show the meaning of Definition 3 and Theorem 1. The universal set of Figure 1 and Figure 2 is the set of outputs $C(P^{\left(K\right)}\left(X\right))$ of states $P^{\left(K\right)}\left(X\right)$ that can reach state *X* after time *K*. Figure 1 shows the state where $C(P^{\left(K\right)}\left(X_{s}\right))$ is completely contained in $C(P^{\left(K\right)}\left(X_{ns_{p}}\right))$. In this case, there exists $C(P^{\left(K\right)}\left(X_{ns_{p}}\right))$ that takes the same value for any value of $C(P^{\left(K\right)}\left(X_{s}\right))$. Therefore, no matter what value the system’s state is in the secret state, the adversary cannot identify that the system’s state is in the secret state from the observations.

On the other hand, in Figure 2, the yellow part is the value of $C(P^{\left(K\right)}\left(X_{s}\right))$ that is included in $C(P^{\left(K\right)}\left(X_{ns_{p}}\right))$, and the red part is the value that is included in $C(P^{\left(K\right)}\left(X_{s}\right))$ but is not included in $C(P^{\left(K\right)}\left(X_{ns_{p}}\right))$. When the output result is in the yellow part of the figure, as in Figure 1, the adversary cannot determine from the observation that the state of the system is a secret state. However, when the output result is in the red part of the figure, there is no $C(P^{\left(K\right)}\left(X_{ns_{p}}\right))$ that has the same value as $C(P^{\left(K\right)}\left(X_{s}\right))$. Therefore, the adversary can determine from the output result that the state of the system is a secret state. For the above reasons, the condition for a system to satisfy opacity is $C\left(P^{\left(K\right)}\left(X_{s}\right)\right)\subseteq C\left(P^{\left(K\right)}\left(X_{ns_{p}}\right)\right)$.

Furthermore, in Definition 3, consider the case where the state does not belong to the secret set $X_{s}$, i.e., it belongs to the non-secret set $X_{ns}$. In this case, the system state is non-secret and therefore does not constitute sensitive information. Accordingly, there is no need to prevent an adversary from identifying the state, and hence opacity need not be considered for this case.

### 4.3. Verification Method

We can verify the opacity of the system (1) by checking whether $C(P^{\left(K\right)}\left(X_{s}\right))$ and $C(P^{\left(K\right)}\left(X_{ns_{p}}\right))$ satisfy the condition (3) or not. In a general case, $C(P^{\left(K\right)}\left(X_{s}\right))$ and $C(P^{\left(K\right)}\left(X_{ns_{p}}\right))$ can be obtained by computation of polytopes. Then, the condition (3) can be verified by comparing the union of multiple polytopes. A general procedure is given as follows.

**Procedure for verifying *p*-opacity of the system (1):**

**Step 1:** Set $Z:=X_{s}$ and i=1.
**Step 2:** Compute $W_{i}:=\left(A_{i}^{-1}Z-A_{i}^{-1}a_{i}\right)⋂S_{i}$ for each i∈{1,2,…,M}.
**Step 3:** Compute $Z:={⋃}_{i=1}^{M}W_{i}$.
**Step 4:** If i=K, then compute C(Z), i.e., $C(P^{\left(K\right)}\left(X_{s}\right))$ and go to Step 5. Otherwise, set i:=i+1 and go to Step 2.
**Step 5:** Set $Z:=X_{ns_{p}}$ and i=1.
**Step 6:** Compute $W_{i}:=\left(A_{i}^{-1}Z-A_{i}^{-1}a_{i}\right)⋂S_{i}$ for each i∈{1,2,…,M}.
**Step 7:** Compute $Z:={⋃}_{i=1}^{M}W_{i}$.
**Step 8:** If i=K, then computer C(Z), i.e., $C(P^{\left(K\right)}\left(X_{ns_{p}}\right))$ and go to Step 9. Otherwise, set i:=i+1 and go to Step 6.
**Step 9:** If $C\left(P^{\left(K\right)}\left(X_{s}\right)\right)\subseteq C\left(P^{\left(K\right)}\left(X_{ns_{p}}\right)\right)$ holds, then the system (1) is *p*-opaque, otherwise the system (1) is not *p*-opaque.
This procedure can be implemented by a suitable software such as Python 3.10. For high-dimensional systems, computing polytopes is frequently hard. In such cases, interval arithmetic may be used instead of computation of polytopes [34,35]. Although intervals are regarded as an over-approximation of polytopes, computation of intervals is easier than that of polytopes.

When the output is a scalar, the condition (3) can be verified more easily than in a general case by comparing the union of multiple intervals. In this case, $C(P^{\left(K\right)}\left(X_{s}\right))$ and $C(P^{\left(K\right)}\left(X_{ns_{p}}\right))$, p=1,2,…,m can be expressed by the following union of intervals:

$$\begin{array}{cc} C(P^{\left(K\right)}\left(X_{s}\right)) & =\overset{L_{s}}{\underset{i=1}{⋃}}\left[y_{s,\min}^{i},y_{s,\begin{array}{c} \max \end{array}}^{i}\right],\\ C(P^{\left(K\right)}\left(X_{ns_{p}}\right)) & =\overset{L_{ns_{p}}}{\underset{i=1}{⋃}}\left[y_{ns_{p},\min}^{i},y_{ns_{p},\begin{array}{c} \max \end{array}}^{i}\right], \end{array}$$

where the numbers $L_{s}$ and $L_{ns_{p}}$ of intervals can be obtained through computation of polytopes, and depend on the dynamics and the number of modes.

### 4.4. Discussion on Special Case

In this subsection, we discuss the case of linear systems. Consider the following linear system:

(4) $$\left\{\begin{array}{c} x(t+1)=Ax(t),\\ y(t)=Cx(t), \end{array}\right.$$

where $x\left(t\right)\in X\subset R^{n}$ is state, $y\left(t\right)\in R^{p}$ is output, and t=0,1,2,… is discrete time. In the case of linear systems, assuming that *A* is invertible, Pre(Z) in (2) is simplified as

$$Pre\left(Z\right):=A^{-1}Z⋂X.$$

In linear systems, the set $P^{\left(K\right)}\left(Z\right)$ is given by (2). In a similar way to Definition 4, we define the notion of opacity for the system (4).

**Definition** **4.** *For the system* (4)*, assume that a set of secret states $X_{s}$ and sets of non-secret states $X_{ns_{p}}$, p=1,2,3,…,m are given. Suppose that the positive constant K is given. Suppose also that an adversary knows the coefficient matrices A,C of the system* (4) *and the output y(t−K) at time t−K, where t is the current time. Then, the system* (4) *is said to be p-opaque if for any state $x\left(t\right)=x^{\prime }\in X_{s}$, there exists an $x\left(t\right)=x^{\prime \prime }\in X_{ns_{p}}$ such that $y^{\prime }\left(t-K\right)=y^{\prime \prime }\left(t-K\right)=y\left(t-K\right)$, where $y^{\prime }\left(t-K\right)$ denotes the output at time t−K for $x\left(t\right)=x^{\prime }\in X_{s}$, and $y^{\prime \prime }\left(t-K\right)$ denotes the output at time t−K for $x\left(t\right)=x^{\prime \prime }\in X_{ns_{p}}$.*

From Theorem 1, we can immediately obtain the following theorem.

**Theorem** **2.** *The system* (4) *is p-opaque if and only if $C\left(P^{\left(K\right)}\left(X_{s}\right)\right)\subseteq C\left(P^{\left(K\right)}\left(X_{ns_{p}}\right)\right)$ holds.*

Initial-state opacity has been studied in [25]. In this paper, we consider the current state opacity. Hence, Theorem 2 is also a new result.

For switched linear systems, based on the result in [29], we can consider the notion of the current state opacity by introducing allowed switching sequences. The details are one of the future challenges.

## 5. Numerical Example

In this section, we demonstrate the effectiveness of the proposed opacity verification method through a numerical example. First, we describe the problem setting and then present the computational results.

Consider the DT-PWA system with nine modes, three states $x\left(t\right)={\left[x_{1}\left(t\right)\;x_{2}\left(t\right)\;x_{3}\left(t\right)\right]}^{⊤}$, and a single output. Coefficient matrices $A_{i}$, i=1,2,…,9 are given as follows:

$$\begin{array}{cc} A_{1} & =\left[\begin{array}{ccc} 0.99 & 0 & 0\\ 0 & 0.7 & 0\\ 0 & 0 & 0.96 \end{array}\right],\;\;A_{2}=\left[\begin{array}{ccc} 0.98 & 0 & 0.1\\ 0.8 & 0.95 & 0\\ 0.1 & 0.2 & 0.9 \end{array}\right],\;\;A_{3}=\left[\begin{array}{ccc} 0.8 & 0 & 0\\ 0 & 0.85 & 0\\ 0 & 0 & 0.9 \end{array}\right],\\ A_{4} & =\left[\begin{array}{ccc} 0.9 & 0 & 0.2\\ 0 & 0.88 & 0\\ 0 & 0 & 0.91 \end{array}\right],\;\;A_{5}=\left[\begin{array}{ccc} 0.8 & 0 & 0\\ 0 & 0.92 & 0\\ 0 & 0 & 0.6 \end{array}\right],\;\;A_{6}=\left[\begin{array}{ccc} 0.89 & 0 & 0\\ 0 & 0.93 & 0\\ 0 & 0.3 & 0.9 \end{array}\right],\\ A_{7} & =\left[\begin{array}{ccc} 0.5 & 0 & 0\\ 0 & 0.95 & 0\\ 0 & 0 & 0.8 \end{array}\right],\;\;A_{8}=\left[\begin{array}{ccc} 0.5 & 0.1 & 0.1\\ 0 & 0.95 & 0.2\\ 0 & 0 & 0.8 \end{array}\right],\;\;A_{9}=\left[\begin{array}{ccc} 0.8 & 0 & 0\\ 0 & 0.92 & 0\\ 0 & 0.3 & 0.9 \end{array}\right]. \end{array}$$

Coefficient vectors $a_{i}$, i=1,2,…,9 are given by

$$\begin{array}{cc} a_{1} & ={\left[\begin{array}{ccc} 0.2 & 0.4 & 0.3 \end{array}\right]}^{⊤},\;\;a_{2}={\left[\begin{array}{ccc} 0.3 & 0.4 & 0.2 \end{array}\right]}^{⊤},\;\;a_{3}={\left[\begin{array}{ccc} 0.2 & 0.1 & 0.2 \end{array}\right]}^{⊤},\\ a_{4} & ={\left[\begin{array}{ccc} 0.1 & 0.2 & 0.3 \end{array}\right]}^{⊤},\;\;a_{5}={\left[\begin{array}{ccc} 0.3 & 0.4 & 0.2 \end{array}\right]}^{⊤},\;\;a_{6}={\left[\begin{array}{ccc} 0.2 & 0.3 & 0.2 \end{array}\right]}^{⊤},\\ a_{7} & ={\left[\begin{array}{ccc} 0.1 & 0.2 & 0.1 \end{array}\right]}^{⊤},\;\;a_{8}={\left[\begin{array}{ccc} 0.2 & 0.1 & 0.1 \end{array}\right]}^{⊤},\;\;a_{9}={\left[\begin{array}{ccc} 0.2 & 0.2 & 0.1 \end{array}\right]}^{⊤}. \end{array}$$

The output matrix *C* is given by C=[100]. That is, the information that the adversary can observe is the value of $x_{1}$. The set of the state is given by

$$X=\left\{{\left[x_{1}\;x_{2}\;x_{3}\right]}^{⊤}\in R^{3}|-10\leq x_{i}\leq 10\right\}.$$

The subsets $S_{i}$ assigned to mode *i*, i=1,2,…,9 are given by

$$\begin{array}{cc} S_{1} & =\left\{x\in X|3<x_{2}\leq 10,\;3<x_{3}\leq 10\right\},\\ S_{2} & =\left\{x\in X|-10\leq x_{2}\leq -3,\;3<x_{3}\leq 10\right\},\\ S_{3} & =\left\{x\in X|-10\leq x_{2}\leq -3,\;-10\leq x_{3}\leq -3\right\},\\ S_{4} & =\left\{x\in X|3<x_{2}\leq 10,\;-10\leq x_{3}\leq -3\right\},\\ S_{5} & =\left\{x\in X|-3<x_{2}\leq 3,\;-3<x_{3}\leq 3\right\},\\ S_{6} & =\left\{x\in X|-3<x_{2}\leq 3,\;-10\leq x_{3}\leq -3\right\},\\ S_{7} & =\left\{x\in X|3<x_{2}\leq 10,\;-3<x_{3}\leq 3\right\},\\ S_{8} & =\left\{x\in X|-3<x_{2}\leq 3,\;3<x_{3}\leq 10\right\},\\ S_{9} & =\left\{x\in X|-10\leq x_{2}\leq -3,\;-3<x_{3}\leq 3\right\}. \end{array}$$

We explain the problem setting about the opacity. Suppose that the set of secret sets $X_{s}$ and the sets of non-secret states $X_{ns_{p}}$, p=1,2,…,8 are given by

$$\begin{array}{cc} X_{s} & =S_{9},\\ X_{ns_{1}} & =S_{1},X_{ns_{2}}=S_{2},X_{ns_{3}}=S_{3},X_{ns_{4}}=S_{4},\\ X_{ns_{5}} & =S_{5},X_{ns_{6}}=S_{6},X_{ns_{7}}=S_{7},X_{ns_{8}}=S_{8}. \end{array}$$

**Figure 3:**
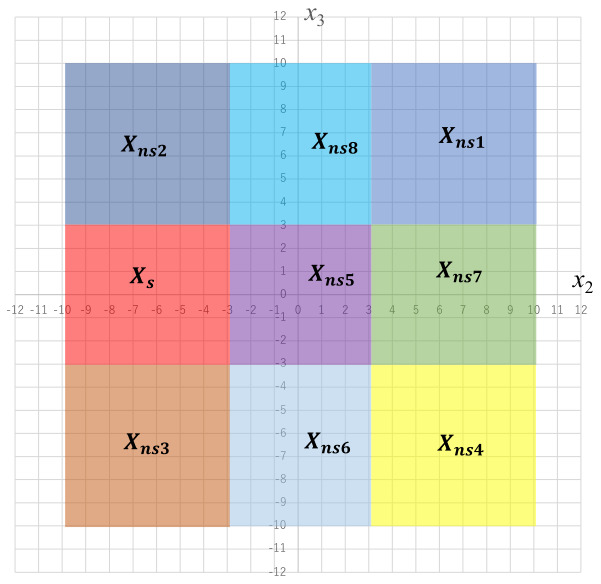
$X_{s}$ and $X_{ns_{p}}$, p=1,2,…,9.

See also Figure 3. Suppose also that the parameter *K* in Definition 3 is given by K=2.

**Figure 4:**
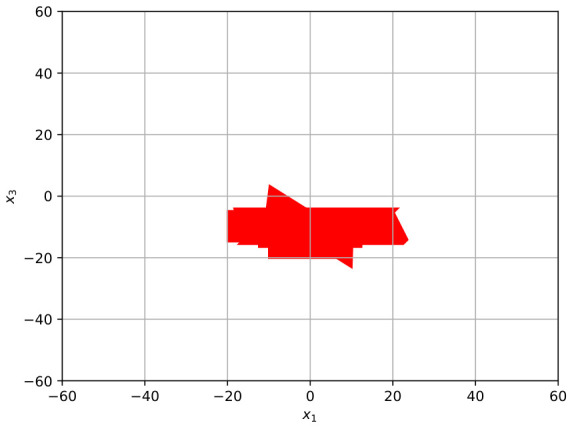
The projection onto the $x_{1}$-$x_{3}$ plane of $P^{\left(2\right)}\left(X_{s}\right)$.

**Figure 5:**
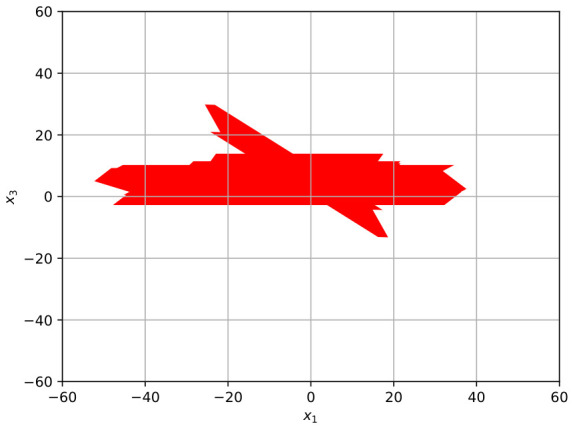
The projection onto the $x_{1}$-$x_{3}$ plane of $P^{\left(2\right)}\left(X_{ns_{1}}\right)$.

**Figure 6:**
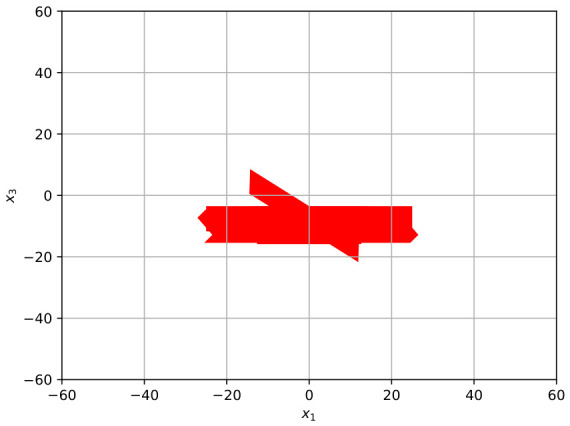
The projection onto the $x_{1}$-$x_{3}$ plane of $P^{\left(2\right)}\left(X_{ns_{2}}\right)$.

**Figure 7:**
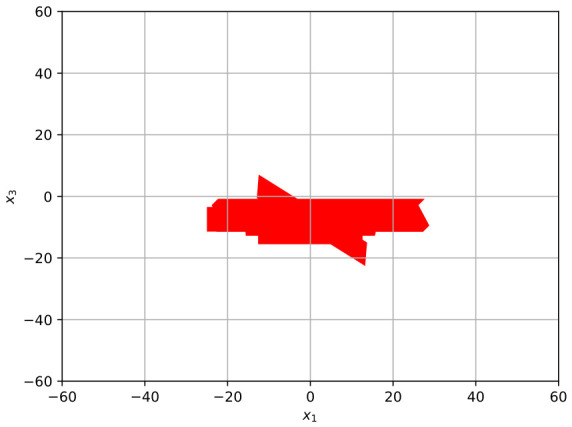
The projection onto the $x_{1}$-$x_{3}$ plane of $P^{\left(2\right)}\left(X_{ns_{3}}\right)$.

**Figure 8:**
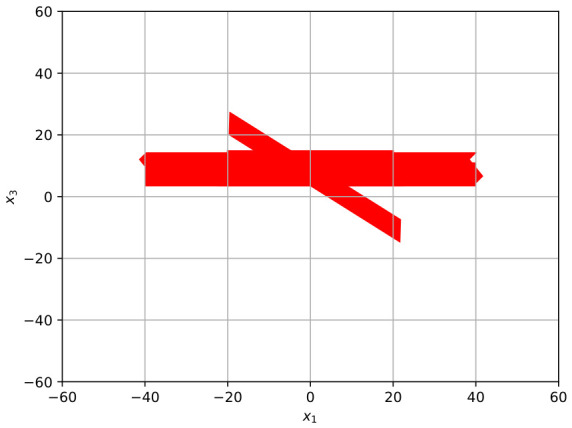
The projection onto the $x_{1}$-$x_{3}$ plane of $P^{\left(2\right)}\left(X_{ns_{4}}\right)$.

**Figure 9:**
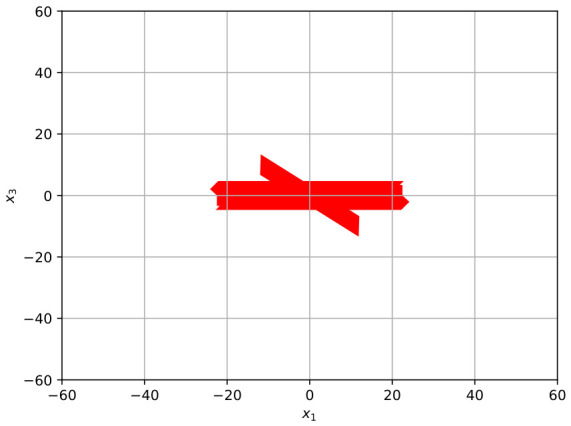
The projection onto the $x_{1}$-$x_{3}$ plane of $P^{\left(2\right)}\left(X_{ns_{5}}\right)$.

**Figure 10:**
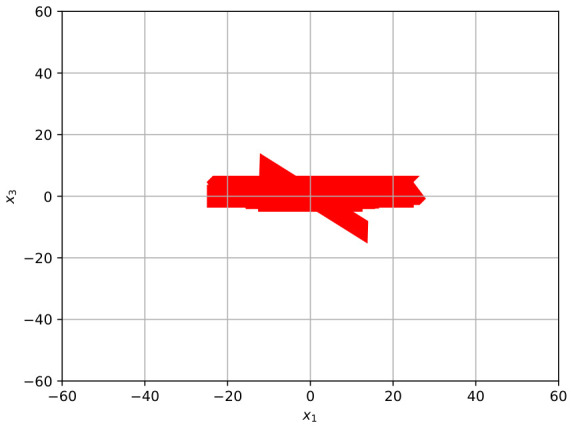
The projection onto the $x_{1}$-$x_{3}$ plane of $P^{\left(2\right)}\left(X_{ns_{6}}\right)$.

**Figure 11:**
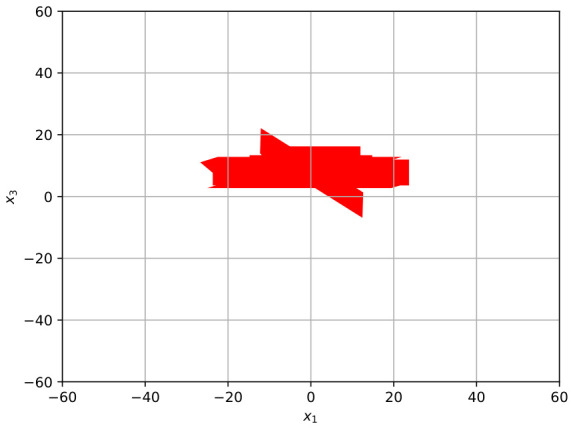
The projection onto the $x_{1}$-$x_{3}$ plane of $P^{\left(2\right)}\left(X_{ns_{7}}\right)$.

**Figure 12:**
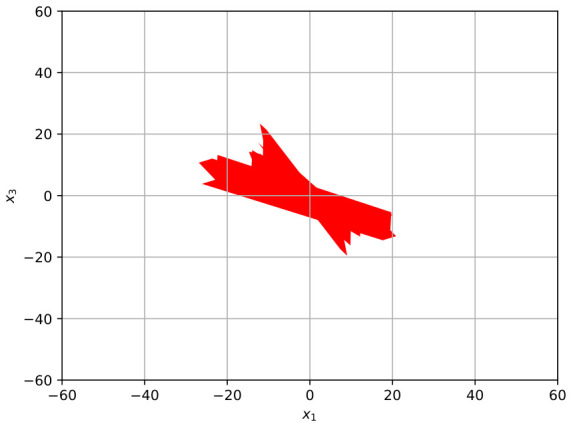
The projection onto the $x_{1}$-$x_{3}$ plane of $P^{\left(2\right)}\left(X_{ns_{8}}\right)$.

Next, we present the computation results. Figure 4, Figure 5, Figure 6, Figure 7, Figure 8, Figure 9, Figure 10, Figure 11 and Figure 12 show the projection onto the $x_{1}$-$x_{3}$ plane of $P^{\left(2\right)}\left(X_{s}\right)$ and $P^{\left(2\right)}\left(X_{ns_{i}}\right)$, i=1,2,…,8. From these figures, $C(P^{\left(K\right)}\left(X_{s}\right))$ and $C(P^{\left(K\right)}\left(X_{ns_{p}}\right))$, p=1,2,…,8 are derived as follows:

$$\begin{array}{cc} C(P^{\left(2\right)}\left(X_{s}\right)) & =[-20.20,23.83],\\ C(P^{\left(2\right)}\left(X_{ns_{1}}\right)) & =[-52.29,37.75],\\ C(P^{\left(2\right)}\left(X_{ns_{2}}\right)) & =[-27.12,26.49],\\ C(P^{\left(2\right)}\left(X_{ns_{3}}\right)) & =[-25.00,28.77],\\ C(P^{\left(2\right)}\left(X_{ns_{4}}\right)) & =[-41.48,41.80],\\ C(P^{\left(2\right)}\left(X_{ns_{5}}\right)) & =[-24.14,24.18],\\ C(P^{\left(2\right)}\left(X_{ns_{6}}\right)) & =[-25.09,27.94],\\ C(P^{\left(2\right)}\left(X_{ns_{7}}\right)) & =[-26.73,23.69],\\ C(P^{\left(2\right)}\left(X_{ns_{8}}\right)) & =[-26.89,20.88]. \end{array}$$

From the above results, we can conclude the following facts:

(i) The system considered in this example is *p*-opaque (p=1,2,3,4,5,6).
(ii) The system considered in this example is not *p*-opaque (p=7,8).

Thus, whether the system is opaque or not depends on the selection of the set of non-secret states.

## 6. Conclusions

In this paper, we proposed a notion of opacity for DT-PWA systems. We derived a necessary and sufficient condition for a system to be *p*-opaque and presented a verification method based on this condition. Through a numerical example, we demonstrated the effectiveness of the proposed method. The results indicate that the proposed framework provides a useful approach to security verification for CPSs. As explained in Section 3.2, the security levels in this paper characterize only the order of subsets of the state space. Therefore, when the proposed method is applied to a real system, it is important to carefully consider the partitioning of the state space and assign security levels to subsets, depending on the characteristics of a given real system.

One of the future efforts is to further analyze the proposed notion of opacity from a theoretical viewpoint. For example, it is important to analyze how the number of polytopes grows with *K* and the number of modes. In addition, future work will address the design of control methods for enforcing opacity [36,37,38,39,40,41].

We will also investigate applications of the proposed method to practical systems such as automotive systems. As an example related to CPSs, we consider adaptive cruise control (ACC). ACC has both physical components (e.g., actuators such as the engine and brakes) and cyber components (e.g., control algorithms). In ACC, modes of a DT-PWA model can correspond to driving modes such as cruise control and vehicle-following control [42,43,44]. In such settings, it is desirable to prevent attackers from inferring the current driving mode related to autonomous driving operations. The dynamics of the driving system in ACC can be modeled as a DT-PWA system in approximately two dimensions [42,43]. Hence, we expect that the proposed notion of opacity can be applied to ACC.

## Data Availability

Data is contained within the article.
